# Enhancing disease awareness for tuberous sclerosis complex in patients with radiologic diagnosis of renal angiomyolipoma: an observational study

**DOI:** 10.1186/s12882-021-02253-w

**Published:** 2021-01-31

**Authors:** Kathrin Bausch, Christian Wetterauer, Julian Diethelm, Jan Ebbing, Daniel T. Boll, Patricia Dill, Cyrill A. Rentsch, Hans-H. Seifert

**Affiliations:** 1 Department of Urology, University Hospital Basel, Spitalstrasse 21, CH-4031 Basel, Switzerland; 2 University of Basel, Petersplatz 1, CH-4051 Basel, Switzerland; 3 Department of Radiology, University Hospital Basel, Petersgraben 4, CH-4031 Basel, Switzerland; 4 Division of Pediatric Neurology and Developmental Medicine, University Childrens’ Hospital Basel, Spitalstrasse 33, CH-4056 Basel, Switzerland

**Keywords:** Angiomyolipoma, Chronic kidney disease, Renal angiomyolipoma, Tuberous sclerosis, Tuberous sclerosis complex

## Abstract

**Background:**

Tuberous Sclerosis Complex (TSC) is a genetic disorder, with renal manifestations like angiomyolipoma (AML) occurring in 70–80% of patients. AML usually cause more complications in TCS patients than in non-TSC patients. However, AML patients are not routinely investigated for TSC. Our aim was to retrospectively assess the correlation between radiologically diagnosed AML and TSC.

**Methods:**

All patients were stratified into AML related vs. unrelated to TSC. Correlations were calculated to determine the association between age, AML, and TSC.

**Results:**

Complete data were available for 521 patients with renal AML, in 7 of which the concurrent diagnosis of TSC was found. Younger age significantly positively correlated with the prevalence of TSC in AML patients (*p* <  0.01). 37 (7%) of the 521 patients were within the age-range of 18–40 years, in which TSC occurred in 6 cases, 4 (66.7%) of which presented with multiple, bilateral renal AML (*p* <  0.05), and 2 (33.3%) of which with a single, unilateral AML (*p* <  0.05). In patients with AML but without TSC, unilateral AML was found in 83.9% and bilateral AML in 16.1% (p <  0.05). Simple binary logistic regression analysis revealed bilateral AML (OR 33.0; 95% CI 3.2–344.0; *p* = 0.003) (but not unilateral AML (OR 0.09; 95% CI 0.01–0.88; *p* = 0.04)) to be a risk factor for TSC.

**Conclusions:**

The presence of bilateral AML in patients within the age-range of 18–40 years should raise suspicion for TSC as the underlying cause. Therefore, our advice is to refer patients with multiple bilateral renal AML for further investigations regarding TSC.

## Background

Tuberous Sclerosis Complex (TSC) is an autosomal dominant genetic disorder with a birth incidence of 1:6000 and an estimated 1 million affected individuals worldwide [1]. TSC is caused by an inactivating mutation in either the TSC-1 or the TSC-2 gene, which code for the proteins hamartin and tuberin [2]. These proteins form a complex that activates the GTPase-activating protein Rheb to inhibit the mechanistic Target of Rapamycin (mTOR). The lack of mTOR-inactivation leads to an increase of protein synthesis, cellular metabolism, differentiation, and growth [3], which may affect virtually every organ. Therefore, TSC is very variable in its clinical manifestations [4]. Formerly, TSC was defined by Vogt’s triad of facial angiofibromas, mental retardation, and intractable epilepsy [5]. Nowadays, the clinical diagnosis is usually confirmed by either a set of major and minor diagnostic criteria or by the identification of a heterozygous pathogenic variant in TSC-1 or TSC-2 by molecular genetic testing [6]. Even though neurological (90%) and cutaneous (90%) manifestations, such as cortical tubers, epilepsy, giant cell astrocytoma, hydrocephalus, neurodevelopmental impairment, and facial angiofibromas [4], are the most common symptoms of TSC, 70 to 80% of TSC patients are also affected by renal angiomyolipoma (AML). Histologically, these benign tumours are composed of blood vessels, adipose tissue, and smooth muscle [7]. In contrast to sporadic AML, TSC-associated AML usually manifest in childhood and adolescence [4, 8]. Growing renal AML pose a significant cause of mortality in TSC patients, since they bear the risk of severe life-threatening haemorrhage [9] and impair the renal parenchyma, which leads to chronic kidney disease (CKD) and eventually end-stage renal disease. Given the current world population, the incidence of TSC, and the frequency of renal involvement, approximately 500,000 patients with TSC worldwide have at least CKD stage 1 [10]. A literature review supports the impression that AML in patients with TSC tends to present earlier, with larger tumours of greater multiplicity, and more frequently in combination with haemorrhage, than in sporadic-type AML patients [11]. In general, surgical resection is avoided whenever possible in order to preserve renal function; interventions are required in case of persistent pain or acute or repeated bleeding episodes. The risk of bleeding increases with the size of AML. Therefore, beyond therapeutic nephron-sparing surgery or embolization, prophylactic intervention in large AML are recommended [12]. In TSC patients, new targeted treatment options with mTOR-inhibitors proved to be beneficial – especially in AML patients [13].

AML are mostly diagnosed by computed tomography (CT), magnetic resonance imaging (MRI), or sonography [14], generally with near certainty due to their unique appearance. An imaging study involving 12,970 male and 4971 female Japanese healthy adults identified renal AML in 13 (0.1%) males and 11 (0.22%) females [15]. However, even though the correlation between AML and TSC has been described and TSC patients have an increased risk for AML-related complications, disease awareness for TSC in AML patients is low in clinical practice.

Therefore, the aim of this study was to investigate whether the radiologic diagnosis of renal AML can lead to the identification of TSC patients and thus increase disease awareness for TSC.

## Methods

### Study design and setting

We performed an observational study at the departments of Urology and Radiology of the University Hospital Basel – a tertiary care centre in Switzerland. We retrospectively analysed all CT, MRI, and sonography reports performed between 2010 and 2016.

The study protocol was approved by the Ethics Committee of Northwestern and Central Switzerland, with a waiver for individual informed consent (No. 2018–00037). We followed the ‘Strengthening the Reporting of Observational Studies in Epidemiology’ guidelines.

### Patient selection

Between January 2010 and December 2016, all CT, MRI, and sonography reports provided by the radiological reporting system were screened using the search terms “AML” and “angiomyolipoma”, in order to identify patients suffering from AML. The occurrence of renal AML was confirmed by manually reviewing the radiologic reports. In case of repeated exams, the exam with the first diagnosis was chosen for analysis. Only patients with completed datasets and patients aged 18 years and older were included.

### Data collection and definitions

We extracted all relevant information from in-house electronic medical records. The medical history was reviewed and patients with previously diagnosed, concurrent TSC (TSC+) were identified by clinical diagnosis in the medical history, independent of the method of initial testing for TSC (e.g. genetic testing, clinical presentation).

### Statistical analysis

Correlations were calculated using a non-normality Spearman’s rank correlation coefficient to determine the association between age, AML, and TSC. Correlation of nominal parameters (age group, AML, and TSC) were calculated using the phi-coefficient. All tests were performed at a significance level of α = 0.05. Continuous data are shown as median with interquartile range (IQR). Simple binary logistic regression analysis was performed to predict a relationship between bilateral or unilateral AML and TSC. All analyses were performed with SPSS Statistics 19 (SPSS Inc., Chicago, Illinois, USA) and Graph Pad Prism Version 6.0 (GraphPad Software, La Jolla, California, USA).

## Results

**Fig. 1:**
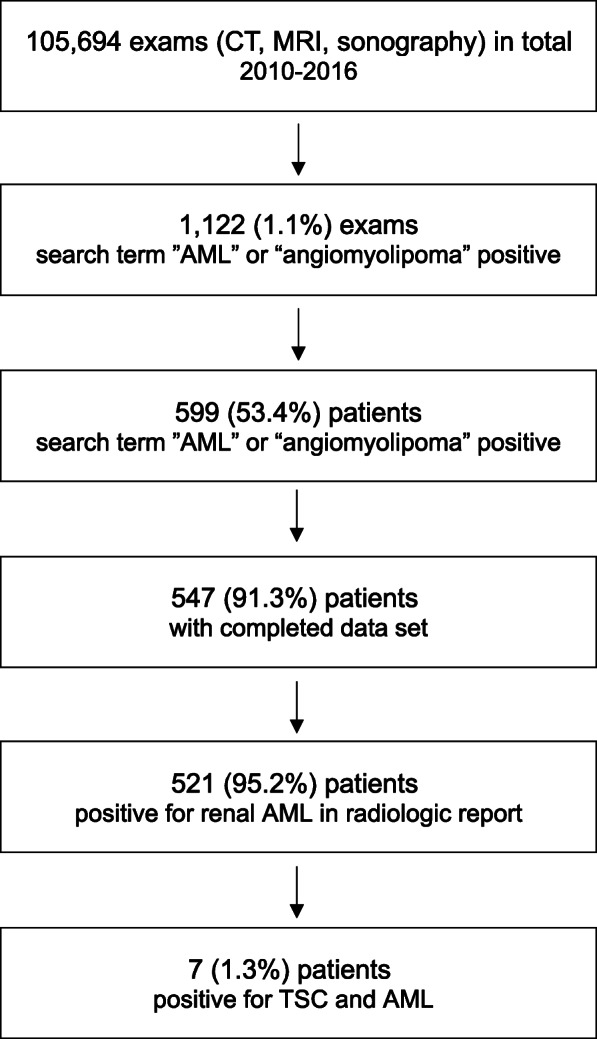
Patient Selection. *AML, angiomyolipoma; CT, computed tomography; MRI, magnetic resonance imaging; TSC, tuberous sclerosis complex*

The search terms were identified in 1122 (1.06%) of the overall 105,694 radiological reports, and the manual review of these reports led to the identification of 599 (53.4%) patients. Complete data were available for 547 (91.3%) of these patients, in 521 (95.2%; 357 (68.5%) females and 164 (31.5%) males) of which renal AML was present (AML+). The concurrent diagnosis of TSC was confirmed in 7 (1.3%) of these 521 patients (3 (0.6%) females and 4 (0.8%) males; Fig. 1).

Median (IQR) AML+ patients’ age was 67.5 (19–84) years, and median (IQR) AML+/TSC+ patients’ age was 28.3 (19–42) years.

AML diagnosis was primarily confirmed by CT (295; 56.6%), followed by sonography (172; 33.0%) and MRI (54; 10.4%) (Table 1).

**Table 1:** Patient Characteristics

	AML+ patients
	(*n* = 521)
*Epidemiology*	
Female, n (%)	357 (68.5)
Male, n (%)	164 (31.5)
Female TSC+, n (%)	3/357 (0.8)
Male TSC+, n (%)	4/164 (2.4)
Age in years, median (IQR)	67.5 (19–104)
Age TSC+ in years, median (IQR)	28.3 (19–42)
18–40 years, n (%)	37 (7.1)
40–45 years, n (%)	15 (2.9)
> 45 years, n (%)	475 (91.2)
*Radiologic examination*	
Sonography, n (%)	172 (33.0)
CT, n (%)	295 (56.6)
MRI, n (%)	54 (10.4)

*AML+, angiomyolipoma positive; CT, computed tomography; MRI, magnetic resonance imaging; TSC+, tuberous sclerosis complex positive*

Among all age groups, we found no significant correlation between presence of AML and concurrent TSC diagnosis (r = 0.03; *p* > 0.553). Younger age, however, significantly correlated with the occurrence of TSC (*p* <  0.01). 37 (7.1%) of the 521 AML+ patients had an age range of 18–40 years, 15 (2.9%) had an age range of 40–45 years, and 475 (91.2%) were older than 45 years (Table 1). Prevalence of TSC in these age range-groups were 16.2% (6/37), 6.7% (1/15), and 0% (0/475), respectively.

**Fig. 2:**
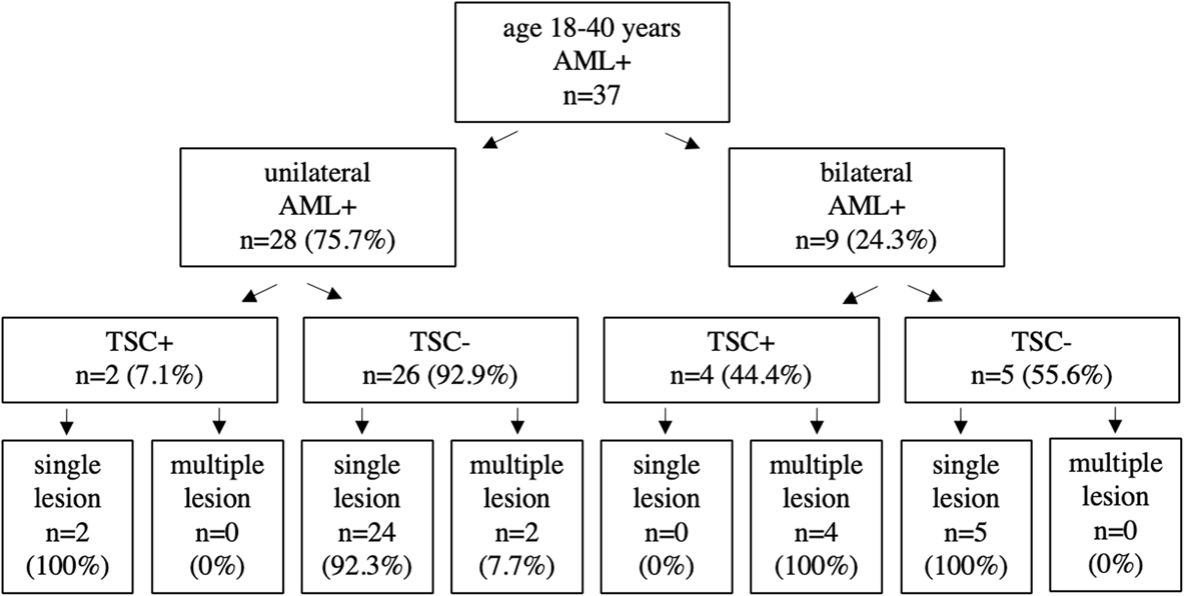
Prevalence of angiomyolipoma and tuberous sclerosis complex in the age group 18 to 40 years. *AML+, angiomyolipoma positive; TSC+/-, tuberous sclerosis complex positive/negative*

The TSC+ patient within the age range of 40–45 years showed multiple bilateral AML. In the age range of 18–40 years, 4 (66.7%) and 2 (33.3%) of the 6 patients presented with bilateral and unilateral renal AML, respectively (*p* <  0.05). TSC+ patients with bilateral renal AML all had multiple AML lesions, whereas all TSC+ patients with unilateral AML had only a single lesion (Fig. 2). In AML+/TSC- patients, unilateral and bilateral AML was found in 83.9% (26/31) and 16.1% (5/31; *p* < 0.05), respectively. Among the TSC- group, unilateral AML presented predominantly (92.3%; 24/26) and bilateral AML uniquely (100%; 5/5) as a single lesion (Table 2, Fig. 2).

**Table 2:** Angiomyolipoma and tuberous sclerosis complex in the age group 18 to 40 years

	AML+	TSC-	TSC+	*p*-values
unilateral	28	83.9% (26/31)	33.3% (2/6)	< 0.05
bilateral	9	16.1% (5/31)	66.7% (4/6)	< 0.05
total	37	31	6	< 0.05

*AML+, angiomyolipoma positive; TSC+/−, tuberous sclerosis positive/negative*

The presence of renal AML, either uni- or bilateral, significantly correlated with a concurrent TSC diagnosis (*p* = 0.028). Simple binary logistic regression analysis revealed bilateral AML (OR 33.0; 95% CI 3.2–344.0; *p* = 0.003) (but not unilateral AML (OR 0.09; 95% CI 0.01–0.88; *p* = 0.04)) to be a risk factor for the presence of TSC (Table 3).

**Table 3:** Simple binary linear regression of angiomyolipoma and tuberous sclerosis

variable	OR	95% CI	p-values
unilateral AML	0.09	0.01–0-88	0.04
bilateral AML	33.0	3.2–344	0.003

*AML+, angiomyolipoma positive; CI, confidence interval, OR, odds ratio*

## Discussion

In our study, we identified an overall TSC prevalence in AML+ patients of 1.3%. Two thirds of the AML+ patients were female and one third male (68.5% vs 31.5%). Accordingly, 0.6% of the male and 0.8% of the female AML+ patients had concurrent TSC. These findings are in line with the literature: while the prevalence of TSC was similar in female and male patients, the clinical manifestation – i.e., the prevalence of AML – differed between genders. The higher prevalence of AML in females has been reported previously [8]. Sporadic AML is known to exhibit predominance in female patients as an effect of growth stimulation of oestrogen and progesterone receptors in AML [1].

We found a significant positive correlation between younger age and TSC prevalence in AML+ patients. Epidemiological studies regarding TSC patients show an increase of renal AML manifestation during childhood and adolescence [4]. In our study, 37 AML+ patients were within the age range of 18–40 years, and the prevalence of TSC in this group was 16.2%. Those findings are in line with the results of prior studies, where 1% of all AML patients had concurrent TCS, but 10% of the AML patients within an age range of 18–40 years [1].

A previous longitudinal study demonstrated that 55% of paediatric TSC patients with a mean age of 6.9 years had some type of renal abnormality, and that this proportion even rose to 80% at a mean age of 10.5 years [15]. Renal AML was by far the most common form of involvement. The authors concluded that in TSC, renal involvement begins in infancy and increases with age. Studies have also demonstrated that the incidence of AML in patients with TSC increased with age and that rapid growth occurred in childhood and adolescence, with slower growth into adulthood [16]. Consequently, we expect that if patients < 18 years would have been included in our study, more cases of TSC would have been identified.

In our cohort, 66.7% of the TCS patients with an age range of 18–40 year presented with bilateral renal AML and 33.3% with unilateral renal AML (*p* < 0.05). Multiple lesions were identified in all TSC+ patients with bilateral AML, whereas primarily single lesions were found in both uni- and bilateral AML+/TSC- patients. The distribution of uni- and bilateral AML in TSC patients has already been investigated in the 1980s: 29% of the AML were unilateral, and 71% occurred on both sides [16]. Our findings are also in line with previous findings that demonstrated more bilateral AML and a higher multiplicity in AML patients with TSC than in AML patients without TSC [11].

In the context of AML occurring at young age, other genetic diseases, which form AML less frequently compared to TSC but are nevertheless similar in phenotype (e.g. renal cysts, AML and renal tumors), should also be considered. These include von Hippel-Lindau syndrome [17] and neurofibromatosis type 1 [18].

In our study, the majority of AML (91.2%) was identified in patients under the age of 45 years. A previously published study investigating the AML incidence in TSC patients demonstrated a significant association between increasing age and the incidence of AML [8]. However, in our cohort, no patient above the age of 45 years had been diagnosed with TSC. These findings may indicate that TSC patients are underdiagnosed due to the formerly limited awareness for TSC and lost to follow-up. Furthermore, life expectancy of most TSC patients is limited to young adulthood due to malignancies and unexpected death in epilepsy. The higher incidence of AML in older patients in our cohort might be explained by more frequent radiologic examinations in comparison to younger patients due to increasing comorbidities, and renal AML could have been detected incidentally. The main limitations of our study are the retrospective design and the small sample size.

In summary, bilateral AML could be identified to be a risk factor for the occurrence of TSC. A previous study on histological findings in AML also concluded that pathologists should recognize that the presence of multiple AML is presumptive evidence for the diagnosis of TSC [19].

The risk for haemorrhage from renal AML in patients with TSC is estimated between 25 and 50% [9], and up to 20% of such patients present with a life threatening haemorrhage [20]. Therefore and as recently discussed in the urological community [21], the diagnosis of AML in radiologic examinations should raise the suspicion of TSC as an underlying multi-system disease, imply further investigations, and initiate targeted treatment with mTOR-inhibitors [13].

## Conclusions

The presence of renal AML in patients with an age-range of 18–40 years should raise suspicion for concurrent TSC as the underlying cause. Therefore, particularly patients with bilateral renal AML should be referred for further testing for TSC, especially in light of the recent changes in the management of TSC and the option of a disease targeted therapy with mTOR-Inhibitors [13].

## Data Availability

The datasets used and/or analysed during the current study are available from the corresponding author on reasonable request.

## Abbreviations

AML: Angiomyolipoma
CKD: Chronic Kidney Disease
CT: Computed Tomography
IQR: Interquartile Range
MRI: Magnetic Resonance Imaging
mTOR: mechanistic Target of Rapamycin
OR: Odds Ratio
TSC: Tuberous Sclerosis Complex
